# Small Conductance Ca^2 +^-Activated K^+^ (SK) Channel mRNA Expression in Human Atrial and Ventricular Tissue: Comparison Between Donor, Atrial Fibrillation and Heart Failure Tissue

**DOI:** 10.3389/fphys.2021.650964

**Published:** 2021-04-01

**Authors:** Elisa Darkow, Thong T. Nguyen, Marina Stolina, Fabian A. Kari, Constanze Schmidt, Felix Wiedmann, István Baczkó, Peter Kohl, Sridharan Rajamani, Ursula Ravens, Rémi Peyronnet

**Affiliations:** ^1^Institute for Experimental Cardiovascular Medicine, University Heart Center Freiburg-Bad Krozingen, Freiburg im Breisgau, Germany; ^2^Medical Center and Faculty of Medicine, University of Freiburg, Freiburg im Breisgau, Germany; ^3^Spemann Graduate School of Biology and Medicine (SGBM), University of Freiburg, Freiburg im Breisgau, Germany; ^4^Faculty of Biology, University of Freiburg, Freiburg im Breisgau, Germany; ^5^Genome Analysis Unit, Amgen Research, Amgen Inc., South San Francisco, CA, United States; ^6^Department of Cardiometabolic Disorders, Amgen Research, Amgen Inc., Thousand Oaks, CA, United States; ^7^Department of Cardiovascular Surgery, University Heart Center Freiburg-Bad Krozingen, Freiburg im Breisgau, Germany; ^8^Department of Cardiology, University Hospital Heidelberg, Heidelberg, Germany; ^9^DZHK (German Center for Cardiovascular Research) Partner Site Heidelberg/Mannheim, Heidelberg University, Heidelberg, Germany; ^10^Department of Pharmacology and Pharmacotherapy, University of Szeged, Szeged, Hungary; ^11^CIBSS Centre for Integrative Biological Signalling Studies, University of Freiburg, Freiburg im Breisgau, Germany; ^12^Translational Safety and Bioanalytical Sciences, Amgen Research, Amgen Inc., South San Francisco, CA, United States

**Keywords:** RNA-seq, SK channels, AF marker genes, atria-selective drugs, atrial fibrillation

## Abstract

In search of more efficacious and safe pharmacological treatments for atrial fibrillation (AF), atria-selective antiarrhythmic agents have been promoted that target ion channels principally expressed in the atria. This concept allows one to engage antiarrhythmic effects in atria, but spares the ventricles from potentially proarrhythmic side effects. It has been suggested that cardiac small conductance Ca^2+^-activated K^+^ (SK) channels may represent an atria-selective target in mammals including humans. However, there are conflicting data concerning the expression of SK channels in different stages of AF, and recent findings suggest that SK channels are upregulated in ventricular myocardium when patients develop heart failure. To address this issue, RNA-sequencing was performed to compare expression levels of three SK channels (*KCNN1*, *KCNN2*, and *KCNN3*) in human atrial and ventricular tissue samples from transplant donor hearts (no cardiac disease), and patients with cardiac disease in sinus rhythm or with AF. In addition, for control purposes expression levels of several genes known to be either chamber-selective or differentially expressed in AF and heart failure were determined. In atria, as compared to ventricle from transplant donor hearts, we confirmed higher expression of *KCNN1* and *KCNA5*, and lower expression of *KCNJ2*, whereas *KCNN2* and *KCNN3* were statistically not differentially expressed. Overall expression of *KCNN1* was low compared to *KCNN2* and *KCNN3*. Comparing atrial tissue from patients with AF to sinus rhythm samples we saw downregulation of *KCNN2* in AF, as previously reported. When comparing ventricular tissue from heart failure patients to non-diseased samples, we found significantly increased ventricular expression of *KCNN3* in heart failure, as previously published. The other channels showed no significant difference in expression in either disease. Our results add weight to the view that SK channels are not likely to be an atria-selective target, especially in failing human hearts, and modulators of these channels may prove to have less utility in treating AF than hoped. Whether targeting SK1 holds potential remains to be elucidated.

## Introduction

Treatment of atrial fibrillation (AF) with antiarrhythmic drugs is hampered by the propensity of pharmacological interventions to induce ventricular proarrhythmic effects. Attempts to circumvent this problem have led to the concept of atria-selective drugs that target ion channels expressed only or predominantly in the atria, but not in the ventricles (for recent review see Peyronnet and Ravens, 2019). Presently, four groups of ion channels are considered to be atria-selective, *i.e.*, the ultra-rapidly activating, outward rectifier K^+^ channel, K_v_1.5 (I_Kur_) (Ravens and Wettwer, 2011), the acetylcholine-activated inward rectifier K^+^ channel, K_ir_2.x (I_K,ACh_) (Koumi and Wasserstrom, 1994), the two-pore domain K^+^ (K_2P_) channels, TWIK-1 (tandem of two-pore-domain, weak inward rectifying K^+^-1) channel and TASK-1 (TWIK-related acid-sensitive K^+^) channel (Gaborit et al., 2007; Schmidt et al., 2015), as well as the small conductance Ca^2+^-activated K^+^ (SK) channels, SK1, SK2, and SK3 (Skibsbye et al., 2014). Novel drugs that selectively target each group of these ion channels have been associated with great expectations for a breakthrough in pharmacological therapy of AF. However, the results of clinical trials related to I_Kur_ and I_K,ACh_ inhibitors have been disappointing, due to lack of efficacy or on-target toxicity (Pavri et al., 2012; Walfridsson et al., 2015; Podd et al., 2016; Shunmugam et al., 2018). A clinical trial regarding the efficacy and safety of a (non-selective) TASK-1 channel inhibitor is still ongoing [Doxapram conversion to sinus rhythm (SR) “DOCTOS” study, EudraCT No: 2018-002979-17, Protocol-Code: K620]. For SK-channel inhibition, preclinical research is ongoing (Saljic et al., 2019; Shamsaldeen et al., 2019; Diness et al., 2020), and this atria-selective antiarrhythmic drug target appears to be the only one not yet investigated in patients (Heijman and Dobrev, 2017).

Apamin, a polypeptide bee venom toxin has been shown to selectively inhibit SK channel current in mouse atrial and ventricular myocytes, as well as in human atrial myocytes (Xu et al., 2003). In addition, apamin prolongs action potential (AP) duration to a much larger extent in atrial than in ventricular murine and human myocytes. In these cells, the atria-selective expression of SK2 channels has been confirmed by Western blot and polymerase chain reaction (Xu et al., 2003; Tuteja et al., 2005). Interestingly, already the first description of cardiac SK channels closes with suggesting them as putative target for treating atrial arrhythmias (Xu et al., 2003). However, results from subsequent work are conflicting. Genetic knock-out of the SK2 channel gene in mice leads to marked atrial AP prolongation with no change in ventricle, but knock-out animals exhibit increased susceptibility to AF (Li et al., 2009). Nagy and co-workers could not detect any effect of apamin on either atrial or ventricular AP duration in rat, dog and human, though determination in human atrial tissue was not carried out (Nagy et al., 2009). Importantly, SK channels can form heteromers from which some were reported to be apamin-insensitive, yet sensitive to small molecule inhibitors such as UCL1684 (Hancock et al., 2015; Shamsaldeen et al., 2019), a feature which may explain some apparent discrepancies between studies. Experimental evidence for the involvement of SK channels in electrical remodeling during AF is also equivocal. In human atrial tissue, upregulation (Zhou et al., 2014) or downregulation (Yu et al., 2012; Ling et al., 2013; Skibsbye et al., 2014) of SK channel expression and apamin-sensitive currents in AF have been reported.

The situation becomes even more unclear when recent experimental evidence in animal and human cardiac tissue is taken into account, which suggests that atria-selectivity of SK channel inhibitors could be compromised in certain clinical conditions (Chua et al., 2011; Chang et al., 2013a,b; Hsueh et al., 2013; Bonilla et al., 2014). For example, ventricular expression of SK2 channels is increased in a rat model of cardiac hypertrophy due to thoracic aortic banding (Hamilton et al., 2020).

Given that AF and heart failure (HF) have been shown to be pathological partners (Santhanakrishnan et al., 2016), expression of SK channels in atrial and left ventricular (LV) tissue from donor hearts not suitable for transplantation (no structural cardiac disease) was compared to expression of SK channels in LV tissue from patients with HF due to ischemic (ICM) or dilated cardiomyopathy (DCM), who required a LV assist device. In addition, expression of the SK channels was studied in human atrial tissue from patients with structural cardiac disease with SR or sustained AF. Expression data were analyzed using a next generation sequencing effort. We confirmed atria-selective expression of K_v_1.5, K_ir_3.1, and SK1 channels but not of SK2 and SK3. Our results suggest that SK channels are expressed both in atria and ventricles, and hence should not be considered as an atria-selective target overall.

## Materials and Methods

### Human Heart Samples

Human atrial tissue samples were obtained from patients with SR or AF, undergoing open heart surgery at the University Heart Center Freiburg-Bad Krozingen or at the Clinic for Cardiac Surgery, Heart Center Dresden. These samples were processed *via* the CardioVascular BioBank (CVBB) Freiburg im Breisgau, as approved by the Ethics Committee of the University of Freiburg, Freiburg im Breisgau, Germany (CVBB ethical approval reference 393/16; study approval reference 10719) and the Ethics Committee of the Medical Faculty of the Technical University Dresden, Dresden, Germany (ethical approval No. 15.1/01031/006/2008 and No. EK790799). LV tissue from patients with HF due to ICM or DCM was provided by the Department of Cardiology, University Hospital Heidelberg, Heidelberg, Germany. The study protocol was approved by the Ethics Committee of the Medical Faculty of Heidelberg University, Heidelberg, Germany (S-017/2013). Atrial and LV myocardium from donor hearts not suitable for transplantation was obtained from the University of Szeged, Szeged, Hungary [Study approval by the Medical Research Council Budapest, Scientific and Research Ethics Committee, Budapest, Hungary No. 4991-0/2010-1018EKU (339/PI/010)]. Hearts were not suitable for transplantation because they could not have reached a recipient in time or because no suitable recipient was found within time. Written and informed consent was obtained from all patients and the study was conducted in accordance with the Declaration of Helsinki.

Atrial and LV myocardium from donor hearts was used as control (no structural cardiac disease) and will be referred to as non-diseased henceforth. Furthermore, one patient was affected by both, coronary artery disease (CAD) *and* heart valve disease (HVD) and this tissue sample was grouped under both conditions. From another patient two tissue samples from the right atrial appendage (RAA) were sequenced. In order to stay consistent with the number of samples per patient per sample provenance and based on the principal component analysis (PCA) plot (not shown), one of those samples was withdrawn from analysis (Supplementary Figure 1). According to the guidelines of the European Society of Cardiology, the American Heart Association, the American College of Cardiology, and the Heart Rhythm Society (January et al., 2014; Kirchhof et al., 2016), for AF, one can distinguish between paroxysmal which is self-terminating or cardioverted within 7 days, and sustained AF which includes patients with persistent (lasting > 7 days), long-standing persistent (lasting > 1 year, therapeutically addressed), and permanent (lasting > 1 year, not therapeutically addressed). This classification is widely accepted both in clinical and research settings (Jahangir et al., 2007; Mittal, 2014). In this study, only tissue from patients with sustained AF was included in the AF group. Of note, all patients with AF also had CAD or HVD, *i.e.*, a structural heart disease, as reason for cardiac surgery. Patient characteristics are summarized in Table 1.

**TABLE 1:** Patient characteristics of sequenced samples.

**Tissue provenance**	**Left ventricle**			**Right atrial appendage**				**Left atrium**
**Health status**	**Non-diseased**	**DCM**	**ICM**	**Non-diseased**	**CAD**	**HVD**	**AF**	**Non-diseased**
Structural heart disease	No	Yes	Yes	No	Yes	Yes	Yes	No
Heart rhythm	SR	SR	SR/AF (4/1)	SR	SR	SR	AF	SR
Number of samples	8	5*	5	7	7*	5*	6	6
Sex (♂/♀)	4/4	5/0	5/0	4/3	7/0	5/0	6/0	3/3
Age (mean ± SEM)	59.9 ± 1.8	44.0 ± 4.0	57.4 ± 2.4	60.3 ± 2.0	66.6 ± 4.1	55.8 ± 8.3	67.5 ± 1.7	61.0 ± 2.0

*DCM, dilated cardiomyopathy; ICM, ischemic cardiomyopathy; CAD, coronary artery disease; HVD, heart valve disease; SR, sinus rhythm; AF, atrial fibrillation; ^∗^ after removing one patient, respectively, for the reasons outlined in the text; SEM, standard error of the mean.*

Due to the limited availability of patient tissue samples, studies on human tissue often compare samples form patients with AF or HF to SR. In order to relate better our results to published work, especially results of functional experiments, LV samples with DCM and ICM were pooled as HF and samples from RAA with CAD and HVD were pooled for patients with SR.

### RNA-Sequencing

Total ribonucleic acid (RNA) was extracted from 5 to 15 mg frozen tissue using the RNeasy Mini kit or the RNeasy Micro kit from Qiagen (Germany) following the manufacturer’s protocols. Samples were first reduced to powder using a Spectrum Bessman pulverizer (Thermo Fisher Scientific, United States), chilled in liquid nitrogen, and homogenized in RLT lysis buffer using a TissueLyser LT (Qiagen) with 5 mm stainless steel beads for 2 × 2 min cycles at 30 Hz. After proteinase K treatment for 10 min at 55°C, homogenates were cleared by centrifugation at 10,000 × *g* for 3 min and loaded on RNeasy Mini Spin Columns or RNeasy MinElute Spin Columns (Qiagen). After on-column deoxyribonuclease treatment and washing, RNA was eluted in 20–30 μL ribonuclease free water and stored at −80°C. In total, RNA was extracted from 40 tissue samples from 39 patients and from 25 tissue samples from 9 donor hearts.

RNA samples were quantified using the Qubit HS RNA kit (Thermo Fisher Scientific) with a Qubit 3.0 Fluorometer (Thermo Fisher Scientific), following manufacturer’s instructions. The integrity of the RNA was determined for all samples using an Agilent Bioanalyzer 2100 (Agilent Technologies, United States) with an Agilent RNA 6000 kit (Agilent Technologies). RNA samples with a RNA integrity number > 7.0 were considered intact and < 7.0 were considered degraded. Based on this, 14 samples were not sequenced (Supplementary Figure 1), including 1x non-diseased (LV), 2x DCM (LV), 3x ICM (LV), 1x non-diseased (RAA), 1x CAD (RAA), 1x CAD and HVD (RAA), 1x ICM (RAA), 2x AF (RAA), 2x non-diseased (LA).

Next generation sequencing libraries were prepared from 200 ng total RNA using the Truseq Stranded messenger RNA (mRNA) kit from Illumina (United States) that includes polyA purification. After quality controls, libraries were pooled in equimolar manner and run on Hiseq 4000 (Illumina) using a 2 × 150 base pairs mode (paired-end) to obtain at least 35 million reads per library.

### Sequencing Data Analysis

The RNA-sequencing (RNA-seq) data analysis was carried out using Galaxy platform^1^ (Afgan et al., 2018), following guidelines from the tutorial “Reference-based RNA-Seq data analysis” (Batut et al., 2018, 2020). In short, quality control checks on raw sequence data was performed using FastQC (Andrews, 2010) and MultiQC (Ewels et al., 2016), taking into account the per base sequence quality (Phred score), per sequence quality scores (Phred score), per base sequence content, per sequence GC content, sequence duplication levels and over-represented sequences. Then, Cutadapt (Marcel, 2011) was used to remove adapter sequences. The splice-aware aligner STAR (Dobin et al., 2012) was used to map the RNA-seq reads onto the human reference genome (hg19). The mapping results were visualized by the integrative genome viewer, IGV (Robinson et al., 2011). Thereafter, the strandness of the RNA-seq data (reads mapping to the forward or reverse DNA strand) was estimated using Infer Experiment from the RSeQC (Wang et al., 2012). Gene expression was measured by featureCounts (Liao et al., 2013). At this step, one sample had to be removed from the study due to assignment of only 41% of the reads (Supplementary Figure 1). To compare between samples, gene counts were normalized for sequencing depth and gene length using transcripts per kilobase million (TPM) (Li and Dewey, 2011). The PCA of gene expression was performed using DESeq2 R package (Love et al., 2014).

### Statistical Analysis

**FIGURE 1:**
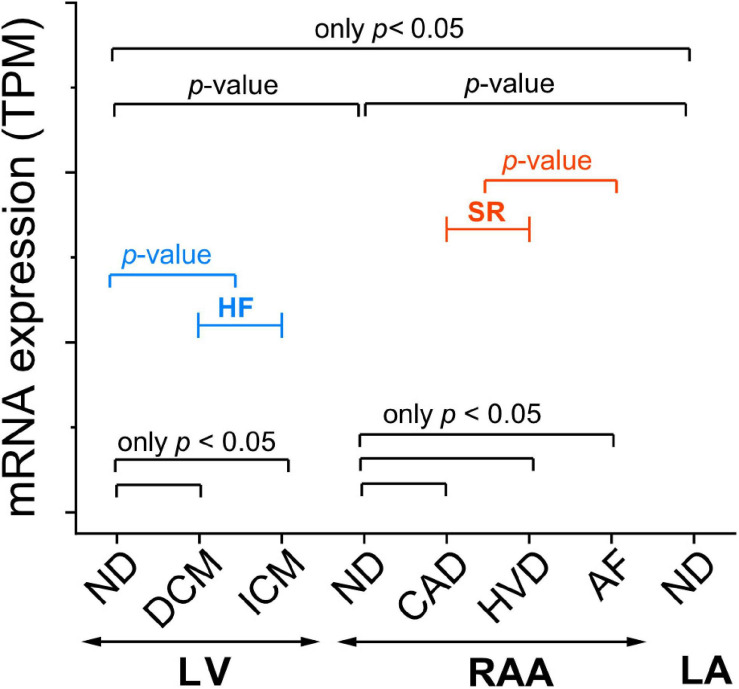
Template figure for presentation of mRNA expression assessed by RNA-seq. Some *p*-values will be indicated in all graphs (*p*-value), whereas others are only indicated when *p* < 0.05 (only *p* < 0.05). Sample provenance: left ventricle (LV), right atrial appendage (RAA), left atrium (LA). Patient’s health status: non-diseased (ND); heart failure (HF; blue) summarizing data from dilated cardiomyopathy (DCM) and ischemic cardiomyopathy (ICM); sinus rhythm (SR; red) summarizing data from coronary artery disease (CAD) and heart valve disease (HVD); atrial fibrillation (AF). Statistical significance was assessed by Dunn’s test. For HF and SR, statistical significance was assessed by the Mann–Whitney test.

Unless otherwise indicated, values are expressed as mean ± standard deviation (SD). N-numbers refer to the number of tissue samples, *i.e.*, number of patients. Significance of the difference between means was assessed using the non-parametric Kruskal–Wallis test and Dunn’s test for *post-hoc* analysis. When pooling conditions into HF and SR, significance of the difference between means was tested with the non-parametric Mann–Whitney test. Figure 1 illustrates the comparisons of interest. A *p*-value < 0.05 was taken to indicate a significant difference between means. Statistical analysis and data representation was performed with OriginPro 2020.

## Results


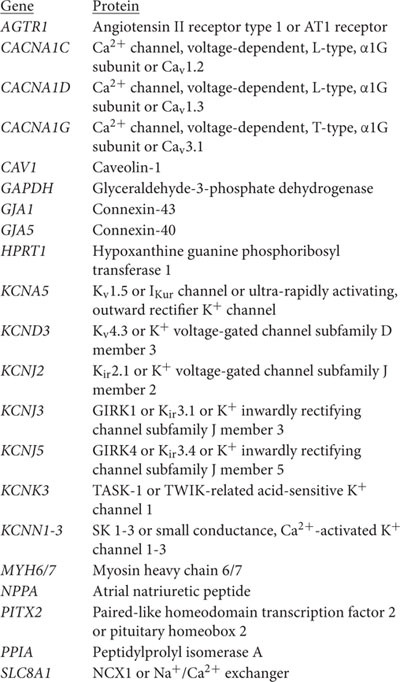


### Sample Characteristics

**FIGURE 2:**
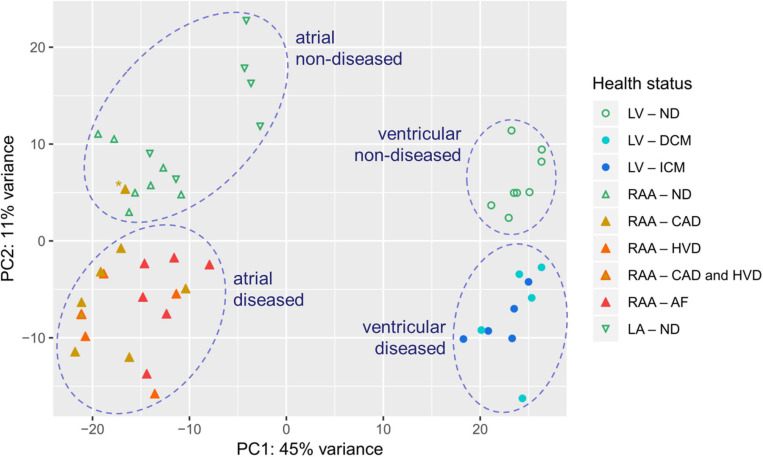
PCA plot illustrating inter- and intra-group variability of samples assessed by RNA-seq. Each individual point represents data from one tissue sample. Dashed circles highlight clusters. ^∗^ indicates a data point showing discrepancy between clinical and experimental data. Sample provenance: left ventricle (LV; •), right atrial appendage (RAA; ▲), left atrium (LA; ▼). Patient’s health status: non-diseased (ND; green, open symbols), dilated cardiomyopathy (DCM; light blue), ischemic cardiomyopathy (ICM; blue), coronary artery disease (CAD; ocher), heart valve disease (HVD; orange), atrial fibrillation (AF; red).

The PCA shows that the first dimension (PC1) roughly separated samples originating from LV from those originating from atria, and the second dimension (PC2) roughly separated samples originating from diseased hearts from those originating from non-diseased hearts (Figure 2). Differential gene expression between atria and LV accounted for 45% of the variability between individual samples, whereas disease-associated differences in gene expression account for 11% of variability. One data point (indicated by ^∗^) corresponded to a patient with CAD and clustered within the non-diseased group. Because of this discrepancy between clinical and experimental data, we decided to exclude this from subsequent analysis (Supplementary Figure 1). One patient was affected by both, CAD *and* HVD. The PCA plot showed that this data point lied within the clusters of these biological conditions and was retained for analysis.

We analyzed the expression of three housekeeping genes as internal controls. Housekeeping genes are defined as genes with a constant expression throughout chambers and throughout health status. We selected the popular *GAPDH* (Molina et al., 2018), *PPIA* (Molina et al., 2018), and *HPRT1* (Cabiati et al., 2012). All three housekeeping genes showed stable mRNA expression between and within heart chambers, indicating a good quality of samples and sequencing (Supplementary Figures 2A–C). Each of these housekeeping genes covered a distinct range of TPM: four-digit range for *GAPDH*, three-digit range for *PPIA*, and one-digit range for *HPRT1*.

### Chamber-Selective mRNA Expression and Modulation Associated With Cardiac Disease

**FIGURE 3:**
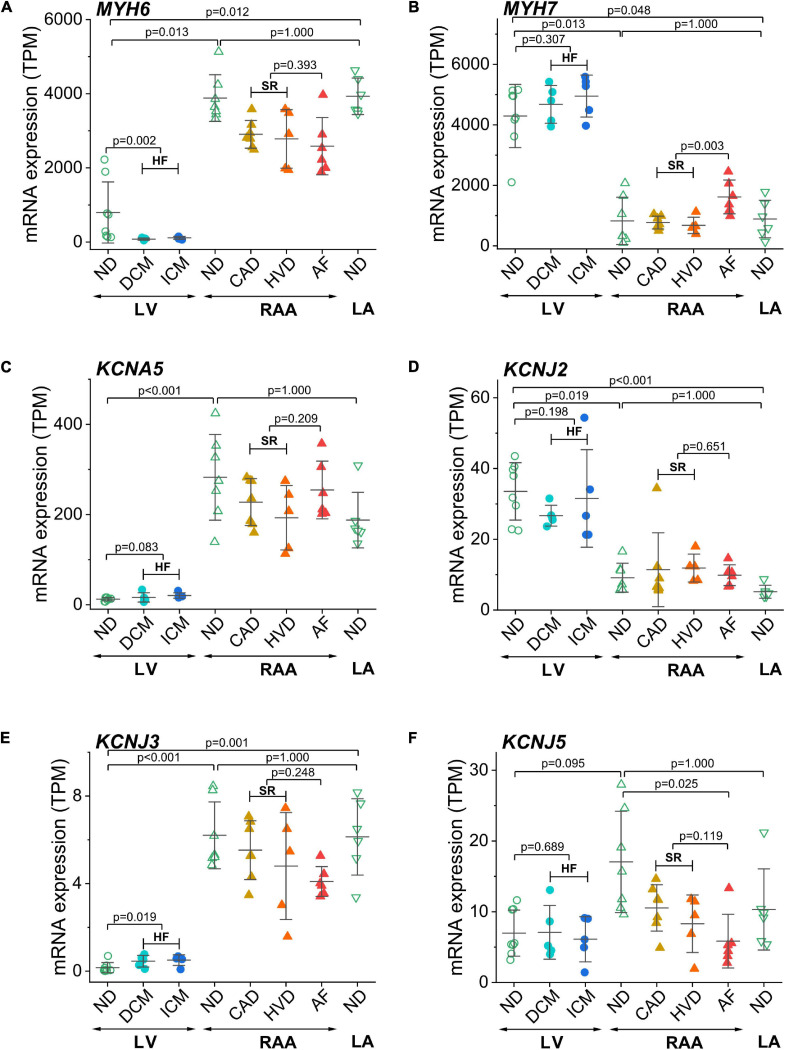
Control of chamber identity and reproducibility of known chamber-selective gene expression in transcripts per kilobase million (TPM) assessed by RNA-seq. **(A)** *MYH6* (myosin heavy chain 6), **(B)** *MYH7* (myosin heavy chain 7), **(C)** *KCNA5* (K_v_1.5 or I_Kur_ channel), **(D)** *KCNJ2* (K_ir_2.1 or K^+^ voltage-gated channel subfamily J member 2), **(E)** *KCNJ3* (GIRK1 or K_ir_3.1 or K^+^ inwardly rectifying channel subfamily J member 3), **(F)** *KCNJ5* (GIRK4 or K_ir_3.4 or K^+^ inwardly rectifying channel subfamily J member 5). Sample provenance and patient’s health status as in Figure 2. Heart failure (HF): pooled data from DCM and ICM; sinus rhythm (SR): pooled data from CAD and HVD. The mean ± SD data are represented. Statistical significance was assessed by Dunn’s test. For HF and SR, statistical significance was assessed by the Mann–Whitney test.

The two isoforms of myosin heavy chain (MHC-α and MHC-β) are encoded by the *MYH6* and *MYH7* genes, respectively. Expression of *MYH6* was dominant in atria, whereas expression of *MYH7* was dominant in the LV, confirming the respective atria- and LV-selective expression of these two genes (Figures 3A,B). Interestingly, in atrial samples from patients with AF, *MYH7* expression was significantly higher than in atrial samples from patients with SR. To validate chamber-selective expression of ion channels, we analyzed the mRNA expression levels of *KCNA5* (encoding K_v_1.5), *KCNJ2* (encoding the inward rectifier K^+^ channel K_ir_2.1), as well as *KCNJ3* and *KCNJ5* (encoding the G-protein-activated inward rectifier K^+^ channels K_ir_3.1 or GIRK1, and K_ir_3.4 or GIRK4, respectively). Expression of *KCNA5* in the LV was low, whereas it was significantly higher RAA tissue, and no differences between non-diseased and diseased heart tissue were detected, neither in atrial nor LV samples (Figure 3C). Expression of the genes encoding inward rectifier K^+^ channels is depicted in Figures 3D–F. For K_ir_2.1, mRNA expression was significantly larger in the LV than in RAA or left atrium (LA), and neither HF nor AF significantly changed the expression level in LV or atria, respectively (Figure 3D). In contrast, expression of *KCNJ3* was significantly higher in RAA and LA than in LV tissue and also higher in HF tissue than in non-diseased LV tissue (Figure 3E). Expression of *KCNJ5*, however, was not significantly different between LV and atrial tissue. In addition, atrial expression of *KCNJ5* was not statistically different between samples from patients with SR and AF but compared with RAA non-diseased samples (and also when compared to all RAA samples from patients with SR), *KCNJ5* expression was significantly lower (Figure 3F). These findings by-and-large confirm the atria-selective expression of some characteristic genes.

**FIGURE 4:**
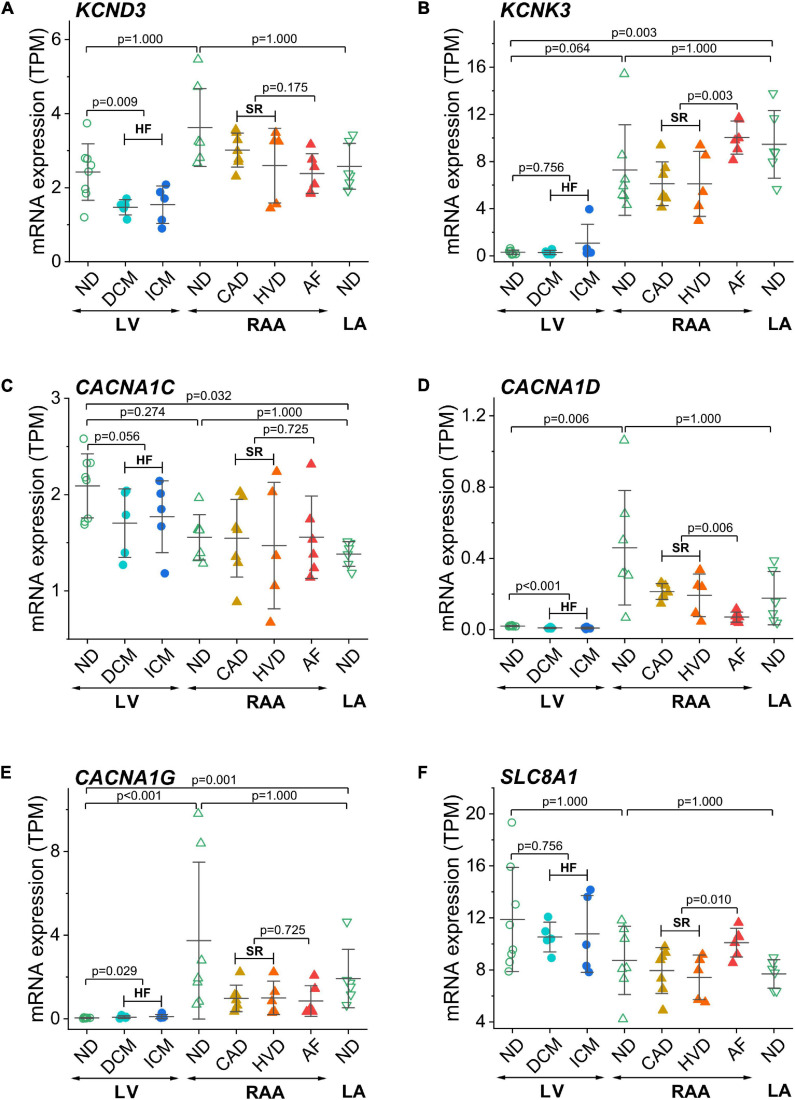
Expression of ion channel encoding genes previously related to AF or HF in transcripts per kilobase million (TPM) assessed by RNA-seq. **(A)** *KCND3* (K_v_4.3), **(B)** *KCNK3* (TASK-1 or TWIK-related acid-sensitive K^+^ channel 1), **(C)** *CACNA1C* (Ca_v_1.2 or Ca^2+^ channel, voltage-dependent, L-type, a1C subunit), **(D)** *CACNA1D* (Ca_v_1.3 or Ca^2+^ channel, voltage-dependent, L-type, a1D subunit), **(E)** *CACNA1G* (Ca_v_3.1 or Ca^2+^ channel, voltage-dependent, T-type, α1G subunit), **(F)** *SLC8A1* (NCX1). Same layout as in Figure 3.

Furthermore, we show genes encoding for ion channels or exchangers for which HF- or AF-associated modulation of expression has been reported previously (Figure 4). *KCND3* contributes to the transient outward K^+^ current, I_to_, *via* K_v_4.3 channels (Figure 4A). Expression of *KCND3* was not statistically different in LV and atria but was lower in LV samples from failing than from non-diseased hearts. *KCNK3* encodes the background K^+^ channel TASK-1 or K_2P_3.1. In contrast to *KCND3, KCNK3* was expressed to a larger extent in LA than LV non-diseased samples and was upregulated in AF (Figure 4B). Cardiac L-type Ca^2+^ channel (Ca_v_1.2 and Ca_v_1.3) genes *CACNA1C* and *CACNA1D* were more extensively expressed in RAA and LA than in the LV, respectively (Figures 4C,D). For *CACNA1C*, no difference was found between non-diseased and diseased tissue samples, whereas *CACNA1D* was downregulated in HF and AF. Expression of the T-type Ca^2+^ channel (Ca_v_3.1) gene *CACNA1G* was higher in RAA and LA than LV non-diseased samples. The low expression of *CACNA1G* in the LV tissue from non-diseased hearts was upregulated in HF samples (Figure 4E). The Na^+^/Ca^2+^ exchanger encoded by *SLC8A1* was statistically not differentially expressed in LV and atrial tissue, but upregulated in AF (Figure 4F).

**FIGURE 5:**
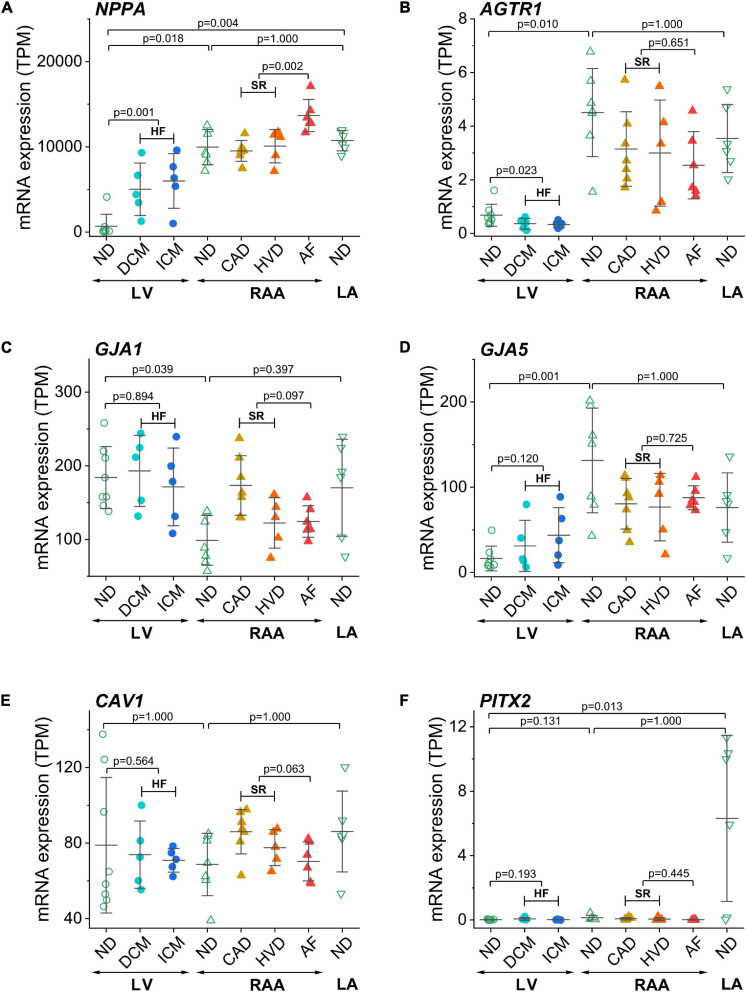
Control of disease identity and reproducibility of known AF- or HF-related gene expression in transcripts per kilobase million (TPM) assessed by RNA-seq. **(A)** *NPPA* (atrial natriuretic peptide A), **(B)** *AGTR1* (AT1 receptor or angiotensin II receptor type 1), **(C)** *GJA1* (connexin-43), **(D)** *GJA5* (connexin-40), **(E)** *CAV1* (caveolin-1), **(F)** *PITX2* (paired-like homeodomain transcription factor 2). Same layout as in Figure 3.

In addition to the above described genes related to ion transport, we also compared the expression of some disease-associated genes (Figure 5). The atrial natriuretic peptide encoding gene *NPPA* was highly abundant in RAA and LA compared to LV. In addition, it was upregulated in both HF and AF (Figure 5A). The angiotensin II receptor type 1 (*AGTR1*) gene was more prominently expressed in RAA than LV non-diseased tissue and when the LV was failing, *AGTR1* expression was significantly lower than in non-diseased LV tissue (Figure 5B). As expected, expression of ventricular connexin-43 encoding gene *GJA1* was more prominent in LV than RAA, whereas the atrial connexin-40 encoding gene *GJA5* was more prominent in RAA (Figures 5C,D). No disease-related modulation of expression was detected in either gene. Another gene whose expression was not statistically different between all conditions was *CAV1* (Figure 5E). *CAV1* encodes for the protein caveolin-1 which is required for forming specialized plasmalemmal invaginations, recruiting and stabilizing large protein complexes involved in signal transduction. The mRNA for transcription factor Pitx2 (paired-like homeodomain transcription factor 2), which is responsible for the development of cardiac left-right asymmetry, was massively expressed in LA non-diseased tissue (Figure 5F).

### Chamber-Selective and Disease-Related mRNA Expression of SK Channels

**FIGURE 6:**
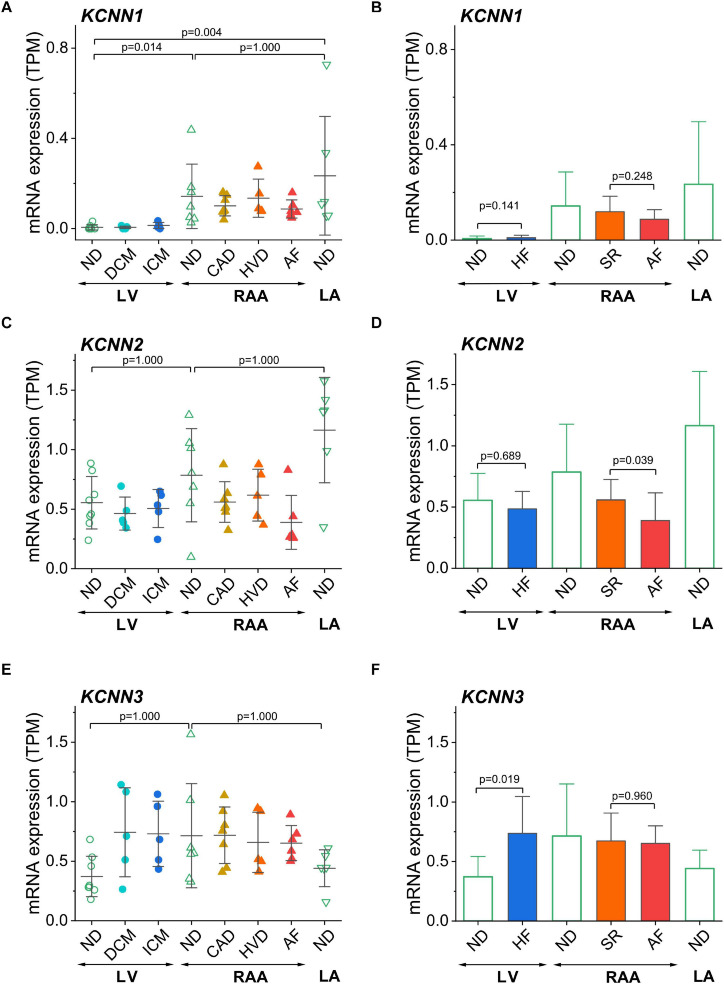
Assessment of chamber-selective and disease-related mRNA expression of SK channels in transcripts per kilobase million (TPM) assessed by RNA-seq. **(A)** Expression of *KCNN1* (SK1) in all sample groups; **(B)** Comparison of *KCNN1* expression in LV tissue from non-diseased and failing hearts, in right atrial tissue from SR and AF patients, and in non-diseased left atrial tissue from non-diseased hearts. **(C,D)** Expression of *KCNN2* (SK2); **(E,F)** expression of *KCNN3* (SK3), figure lay-out as in **(A,B)**. Sample provenance, patient’s health status and statistical tests as in Figure 3. Since disease-associated differences in expression of SK channels were our main concern, we separately depicted the statistical comparisons for HF and AF **(B,D,F)**.

The genes described so far were selected because of their previously reported chamber-selective expression or because of known modulation of expression in HF or AF. Since the main aim of this study was (i) to confirm chamber-selective expression of SK channels and (ii) to comment on the therapeutic potential of these channels in pharmacological treatment of AF, we have analyzed the expression levels of three SK channels encoded by *KCNN1*, *KCNN2*, and *KCNN3* in our set of human cardiac tissue samples (Figure 6). For *KCNN1*, significantly lower mRNA expression was found in non-diseased LV than in non-diseased RAA and LA (Figure 6A), whereas *KCNN2* and *KCNN3* (Figures 6C,E) showed no chamber-selective mRNA expression.

These results put into question the assumed atria-selective expression of SK channels in general. In order to answer the question of changes of ventricular SK channel expression in association with HF, and of atrial SK channel expression in association with AF, we also compared non-diseased LV tissue to all samples from failing hearts, and all diseased RAA samples from patients with SR to RAA tissue from AF patients. Interestingly, of the SK channels, only LV expression of *KCNN3* was higher in HF, whereas *KCNN1* and *KCNN2* were statistically not differentially expressed. When comparing AF *vs.* SR tissue samples, *KCNN1* and *KCNN3* were not statistically different (Figures 6B,F), while *KCNN2* was downregulated (Figure 6D).

## Discussion

In the present study using next generation sequencing, gene expression in three types of human LV and five types of atrial tissue samples was determined. Our main results are: (i) principal component analysis yields clear clustering of atrial *vs.* LV samples, and of diseased *vs.* non-diseased myocardium; (ii) in non-diseased hearts, only SK1 (but not SK2 and SK3) channels are expressed to a significantly larger extent in atrial (RAA and LA) than in LV tissue samples; (iii) HF is associated with an upregulation of SK3 channel, and AF is associated with a downregulation of SK2 isoform. These findings will be discussed below.

### Sample Characteristics

The various myocardial samples had to be collected from different hospitals, depending on tissue availability, but all were processed in the same laboratory for RNA extraction and preparation of the next generation sequencing libraries in order to ensure best possible comparability. Despite the high number of samples in which mRNA extraction was of insufficiently high quality – in total, 14 samples had to be rejected – PCA for all sixty thousand genes tested in the remaining 49 samples yielded good clustering (Figure 2). Indeed, PCA demonstrated four large clusters of data sets separating atrial from LV and non-diseased from diseased heart samples. Only one data point from RAA tissue from a CAD patient fell into the cluster for non-diseased tissue and was discarded from further analysis. The dimensions of the clusters demonstrate the scattering of the data points within a biological condition which is inherently due to inter-individual variability.

### Chamber-Selective mRNA Expression and Modulation Associated With Cardiac Disease

Given the large scatter in values within each sample group, the expression of several additional genes of known chamber-selective expression or previously reported disease-associated modulation was determined for control purposes. *MYH6* and *MYH7* encoding the two MHC isoforms MHC-α and MHC-β were utilized for validation of atria- or LV-selective gene expression (Everett, 1986; Kurabayashi et al., 1988). In accordance with the literature, expression of *MYH6* was significantly higher in atrial than LV tissue, and the opposite was true for *MYH7* expression.

The cardiac K^+^ channel K_v_1.5 is the best studied representative of an atria-selective drug target based on electrophysiological and mRNA expression data (Amos et al., 1996; Gaborit et al., 2007). Numerous small molecule K_v_1.5 inhibitors intended for treatment of AF have been developed (Ford and Milnes, 2008). Our results confirm previously published atria-selective expression of human *KCNA5* and lack of AF-associated remodeling of mRNA expression (Gaborit et al., 2007). Table 2 summarizes the results of all genes that were tested for preferential expression in atria or LV in comparison with the literature. For the vast majority of cases we confirm the reported chamber-selective expression, although in some cases we were not able to detect the reported differences. However, this is not surprising, given the differences in patient numbers and populations as well as the different experimental conditions. Taken together, the chamber-selective expression of control genes reported here is in good agreement with the published literature (as summarized in Table 2) suggesting that our RNA analysis is reliable.

**TABLE 2:** Summary of differential gene expression for all genes in this study.

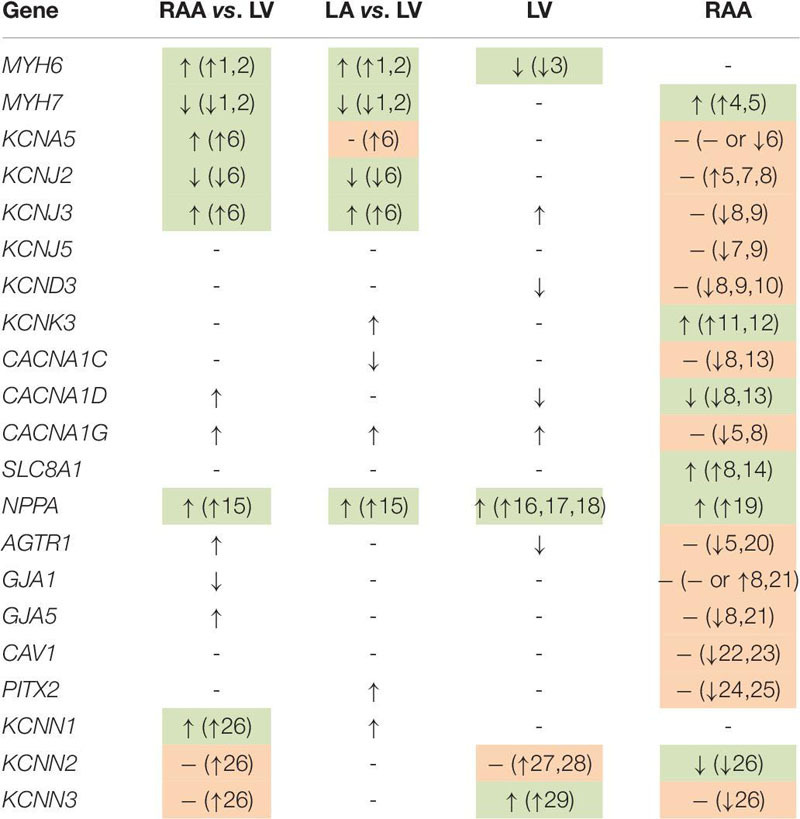

*Upregulation (↑), downregulation (↓), or no significant difference (-) are indicated such that the symbols *outside* parentheses sum up our experimental observations, and the symbol *within* parentheses previously reported findings form the literature. For 21 comparisons our observations match prior reports (green background). In 17 cases, our data did not show statistically significant differences for comparisons that had previously been reported to differ (orange background). Sample provenance: left ventricle (LV), right atrial appendage (RAA), left atrium (LA). Patient’s health status: non-diseased (ND); heart failure (HF): pooled data from dilated cardiomyopathy and ischemic cardiomyopathy; sinus rhythm (SR): pooled data from non-diseased, coronary artery disease and heart valve disease; atrial fibrillation (AF).^1^Everett, 1986;^2^Kurabayashi et al., 1988;^3^Miyata et al., 2000;^4^Nielsen et al., 2018;^5^Thomas et al., 2019;^6^Gaborit et al., 2007;^7^Dobrev et al., 2001;^8^Gaborit et al., 2005;^9^Brundel et al., 2001;^10^Grammer et al., 2000;^11^Schmidt et al., 2015;^12^Schmidt et al., 2017;^13^Van Wagoner and Nerbonne, 2000;^14^Schotten et al., 2002;^15^Macchia, 1987;^16^Chien et al., 1991;^17^Schmitt et al., 2003;^18^Houweling et al., 2005;^19^Büttner et al., 2018;^20^Goette et al., 2000;^21^Nao et al., 2003;^22^Yi et al., 2014;^23^Martin et al., 2015;^24^Chinchilla et al., 2011;^25^Kirchhof et al., 2011;^26^Skibsbye et al., 2014;^27^Chang et al., 2013a;^28^Hamilton et al., 2020;^29^Bonilla et al., 2014.*

Since the focus of this study was not only at chamber-selective but also disease-modulated gene expression, some genes that are known to be differentially expressed in HF or AF, as for instance *NPPA* (Macchia, 1987), have been analyzed. We confirm that expression of *NPPA* was dominant in non-diseased atria, but LV *NPPA* expression was activated in response to HF (Chien et al., 1991; Schmitt et al., 2003; Houweling et al., 2005). Moreover, *NPPA* expression was upregulated in RAA from patients with AF (Büttner et al., 2018). In addition, we also found AF-associated upregulation of *MYH7* (Nielsen et al., 2018; Thomas et al., 2019), *KCNK3* (Schmidt et al., 2015, 2017), *SLC8A1* (Schotten et al., 2002; Gaborit et al., 2005), and downregulation of *CACNA1D* (Van Wagoner and Nerbonne, 2000; Gaborit et al., 2005). For the other genes, for which AF-induced expression changes have been reported (see Table 2), we have not detected any significant changes in expression between atrial samples from patients with AF or SR. However, for two comparisons, part of the published literature agrees with our inability to detect a difference. Not in a single case did we observe statistically significant differences that were in the opposite direction to previously published reports. Failure to detect a significant difference does not indicate absence of any difference, but could also imply that the number of samples (in either of the analyzed cohorts) may not have been large enough. Overall, the agreement of our expression profiles with previous reports instills confidence that – where we do see differences – we can draw reliable conclusions, such as about SK channel expression in human atrial and LV myocardium.

### Chamber-Selective and Disease-Related mRNA Expression of SK Channels

The main purpose of our analysis was to assess whether SK channels are expressed to a much larger extent in atria than in the LV, which would presumably be necessary for them to serve as atria-selective drug targets. The first detection of SK channels in the heart demonstrated greater abundance in atria than ventricles, although only in mouse and not in human (Xu et al., 2003). Since then, evidence for atria-selective expression of SK channels has been obtained for many species (Tuteja et al., 2005: mouse; Diness et al., 2010: rat, guinea pig, rabbit; Skibsbye et al., 2014: human). To the best of our knowledge, our study is the first report of a direct comparison of atrial and LV SK channel expression in human non-diseased and diseased myocardium. We found a significantly higher expression of *KCNN1* in RAA and LA tissue than in LV from non-diseased hearts, suggesting potential atria-selectivity for the SK1 channel. However, despite substantial expression of *KCNN2* and *KCNN3* in all chambers, these genes were not significantly enriched in the atria (compared to LV) in non-diseased hearts. Since non-diseased hearts do not require therapy, and since atria-selective drugs are intended for treatment of AF, we scrutinized SK channel expression in atrial tissue from patients with AF in comparison to atrial tissue from non-diseased donors or patients (CAD, HVD) with SR. In accordance with previous findings for AF, expression of *KCNN1* did not significantly change and *KCNN2* was downregulated, whereas we did not observe the previously reported downregulation of *KCNN3* expression (Skibsbye et al., 2014). The rationale for suppressing atrial arrhythmias with SK channel blockers is to increase atrial refractoriness (Diness et al., 2020). The observed downregulation of SK2 may be an auto-regulatory mechanism which could be promoted by SK channel inhibitors.

Heart failure is a common comorbidity of AF and is associated with remodeling of ion channel expression in the ventricles (Nattel et al., 2007). Therefore, we also compared SK channel expression in tissue from failing and non-diseased human hearts. Of the three SK channels, *KCNN3* was upregulated in failing human LV, while the other two SK channels were not statistically different. In the literature, SK channels in general were reported as being upregulated in failing rabbit hearts (Chua et al., 2011), SK2 channels in human failing (*vs.* non-failing) ventricular tissue or hypertrophied mouse ventricles (Chang et al., 2013a; Hamilton et al., 2020), and SK3 channels in failing dog hearts (Bonilla et al., 2014). It is an ongoing debate of whether SK channel upregulation is anti- or proarrhythmic (Chang and Chen, 2015; Van Wagoner, 2015).

Of course, it is difficult to correlate mRNA expression and protein function. For SK channels, this is further complicated by the fact that most inhibitors or activators are not selective for one of the three subtypes. For example, the most commonly used SK channel inhibitor, apamin, shows only some selectivity between SK channel subtypes, with SK2 being the most sensitive (Weatherall et al., 2011). Furthermore, the physiological role of SK channels in the heart is complex. There is evidence suggesting that in human atrial myocytes, activation of SK2-SK3-heteromers contributes to repolarization of the AP. These SK2-SK3-heteromers are insensitive to apamin, but inhibited by the small organic molecule inhibitor UCL1684 (Hancock et al., 2015; Shamsaldeen et al., 2019). Therefore, one cannot reliably extrapolate from observed mRNA levels to SK channel subtype function.

In any case, though, upregulation of ventricular SK channels such as SK2 and SK3 in HF, in the presence of unchanged or downregulated expression in the atria, would suggest reduced potential for atria-selectivity of pharmacological interventions. Based on our mRNA expression data from human tissue samples, only SK1 channels fulfilled the criteria for an atria-selective drug target that is conserved in HF. However, the contribution of SK1 channels to repolarization of the action potential is not well established because functional data are usually obtained with the SK channel inhibitor apamin – to which SK1 channels are least sensitive (Grunnet et al., 2001). This calls for further basic research, using SK1-targeting genetic or pharmacological interventions, to fully evaluate its potential as an atria-selective target.

### Limitations

The number of biological replicates was low, due to the limited quantity of patient material and the exclusion of several samples with low RNA integrity numbers, however, it was ≥ 5 for each sub-goup, which we regard as a lower limit. Higher n-numbers would be needed to assess the question of whether the here reported lack of statistically significant changes in certain RNA levels previously reported to be up- or downregulated (Table 2), offers a generalizable view of electrophysiological remodeling in AF.

All diseased human hearts samples were from male patients while non-diseased samples came in equal parts from male and female individuals. This is due to the fact that the number of patients was limited and we had to prioritize clinical entities, rather than gender. For this reason, we cannot address sex-specific differences in mRNA expression. Sex-related differences in myocardial gene expression have been reported previously (Guo et al., 2017; Inanloorahatloo et al., 2017), so this forms an area for further research.

Right atrial appendage tissue from AF patients is compared to tissue from patients with SR to describe changes in gene expression in patients with AF. While RAA is the human atrial tissue that is most frequently removed during open heart surgery and therefore available for scientific investigation, analysis of gene expression in the LA would be of even greater interest, as AF is most is often associated with electrophysiological abnormalities in LA and pulmonary veins (Haïssaguerre et al., 1998).

In the past, several groups have searched for altered ion channel expression in human atrial tissue in order to explain the typical changes in AP shapes (electrical remodeling) seen in AF (Van Wagoner and Nerbonne, 2000; Dobrev and Ravens, 2003). Changes in expression of mRNA do not necessarily manifest themselves in matching changes in presence and activity of the proteins they encode. A particular gene product may be further modified during translational or even by post-translational processes. Moreover, newly synthetized proteins such as ion channels can remain trapped in the endoplasmic reticulum for some time before they are recruited to their proper functional compartment, the plasmalemma (Schumacher and Martens, 2010). Therefore, the here presented results do not provide insight into the actual electrophysiological remodeling in AF.

## Conclusion

In this study, we assess key genes with selective expression in either atria or LV, and their up- or downregulation in AF or HF. We document SK channel expression in atria and LV of hearts from patients with or without structural cardiac disease. We conclude that only SK1, but not SK2 and 3, shows a preferential expression in the atria in non-diseased and diseased hearts, albeit at low expression levels whose functional relevance remains to be assessed. General blockers of SK channels are not likely to have atria-selective effects, and hence may prove to have less utility in treating AF than hoped.

## Data Availability Statement

The datasets presented in this study can be found in online repositories. The names of the repository/repositories and accession number(s) can be found below: https://www.ebi.ac.uk/ena/browser/view/PRJEB42485.

## Ethics Statement

The studies involving human participants were reviewed and approved by the Ethics Committee of the University of Freiburg, Freiburg im Breisgau, Germany, the Medical Faculty of the Technical University Dresden, Dresden, Germany, the Medical Faculty of Heidelberg University, Heidelberg, Germany and the Medical Research Council Budapest, Scientific and Research Ethics Committee, Budapest, Hungary. The patients/participants provided their written informed consent to participate in this study.

## Author Contributions

ED, TN, MS, PK, SR, UR, and RP contributed to the conception, design, and interpretation of the study. ED, UR, and RP drafted the manuscript. ED analyzed the RNA-seq data. FK, CS, FW, and IB provided human heart samples. All authors contributed to manuscript revision, read, and approved the submitted version.

## Conflict of Interest

TN, MS, and SR were employed by Amgen Inc., at the time of submission of the manuscript. The remaining authors declare that the research was conducted in the absence of any commercial or financial relationships that could be construed as a potential conflict of interest.

## Footnotes

1 https://usegalaxy.eu/

## Supplementary Material

Supplementary Figure 1 Flow chart illustrating the inclusion criteria. Number (N°) of human heart tissue samples entered, removed after assessment at different quality control stages, and finally included in the analyses is shown. PCA, Principal component analysis; TPM, transcripts per kilobase million.

Supplementary Figure 2 mRNA expression of housekeeping genes in transcripts per kilobase million (TPM) assessed by RNA-seq. **(A)** *GAPDH* (glyceraldehyde-3-phosphate dehydrogenase), **(B)** *PPIA* (peptidylprolyl-isomerase A), and **(C)** *HPRT1* (hypoxanthine-phosphoribosyltransferase 1). Sample provenance: left ventricle (LV; •, right atrial appendage (RAA; ▲), left atrium (LA; ▼). Patient’s health status: non-diseased (ND; green, open symbols); heart failure (HF): pooled data from dilated cardiomyopathy (DCM; light blue) and ischemic cardiomyopathy (ICM; blue); sinus rhythm (SR): pooled data from coronary artery disease (CAD; ocher) and heart valve disease (HVD; orange); atrial fibrillation (AF; red). The mean ± SD data are represented. Statistical significance was assessed by Dunn’s test. For HF and SR, statistical significance was assessed by the Mann–Whitney test.

## Abbreviations

AF: Atrial fibrillation
AP: Action potential
Ca^2+^: Calcium
CAD: Coronary artery disease
CVBB: Cardiovascular biobank
DCM: Dilated cardiomyopathy
GIRK: G protein-coupled inwardly rectifying K^+^ channel
HF: Heart failure
HVD: Heart valve disease
ICM: Ischemic cardiomyopathy
K^+^: Potassium
K_2P_ channels: Two-pore-domain K^+^ channels
LA: Left atrium
LV: Left ventricle
mRNA: Messenger RNA
Na^+^: Sodium
PCA: Principal component analysis
RAA: Right atrial appendage
RNA: Ribonucleic acid
RNA-seq: RNA-sequencing
SD: Standard deviation
SK channel: Ca^2+^-activated K^+^ channel of small conductance
SR: Sinus rhythm
TASK-1 channel: TWIK-related acid-sensitive K^+^ channel 1
TPM: Transcripts per kilobase million
TWIK channels: Tandem of two-pore-domain weak inward rectifying K^+^ channels.
