# Supplementary figures and images for: Small Conductance Ca2 +-Activated K+ (SK) Channel mRNA Expression in Human Atrial and Ventricular Tissue: Comparison Between Donor, Atrial Fibrillation and Heart Failure Tissue
Source: Front Physiol. 2021 Apr 1;12:650964. doi: 10.3389/fphys.2021.650964 (PMC8047327; doi:10.3389/fphys.2021.650964)

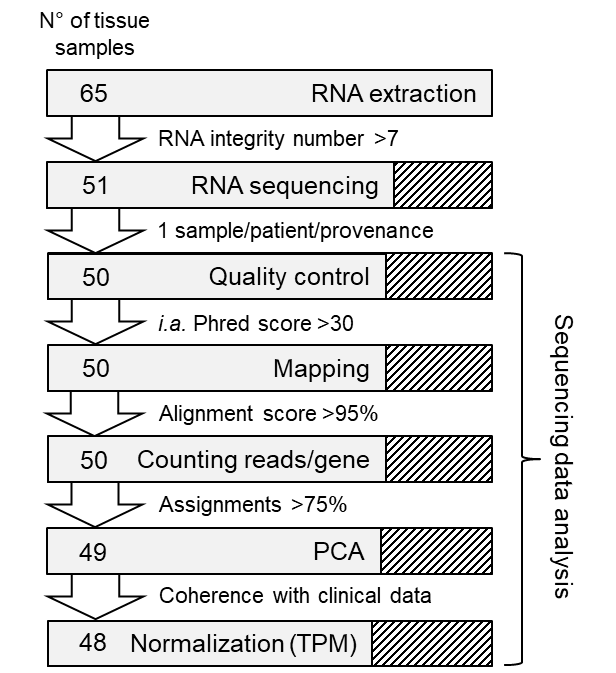

Supplement: Supplementary Figure 1 — Flow chart illustrating the inclusion criteria. Number (N°) of human heart tissue samples entered, removed after assessment at different quality control stages, and finally included in the analyses is shown. PCA, Principal component analysis; TPM, transcripts per kilobase million. [file Image_1.TIF]

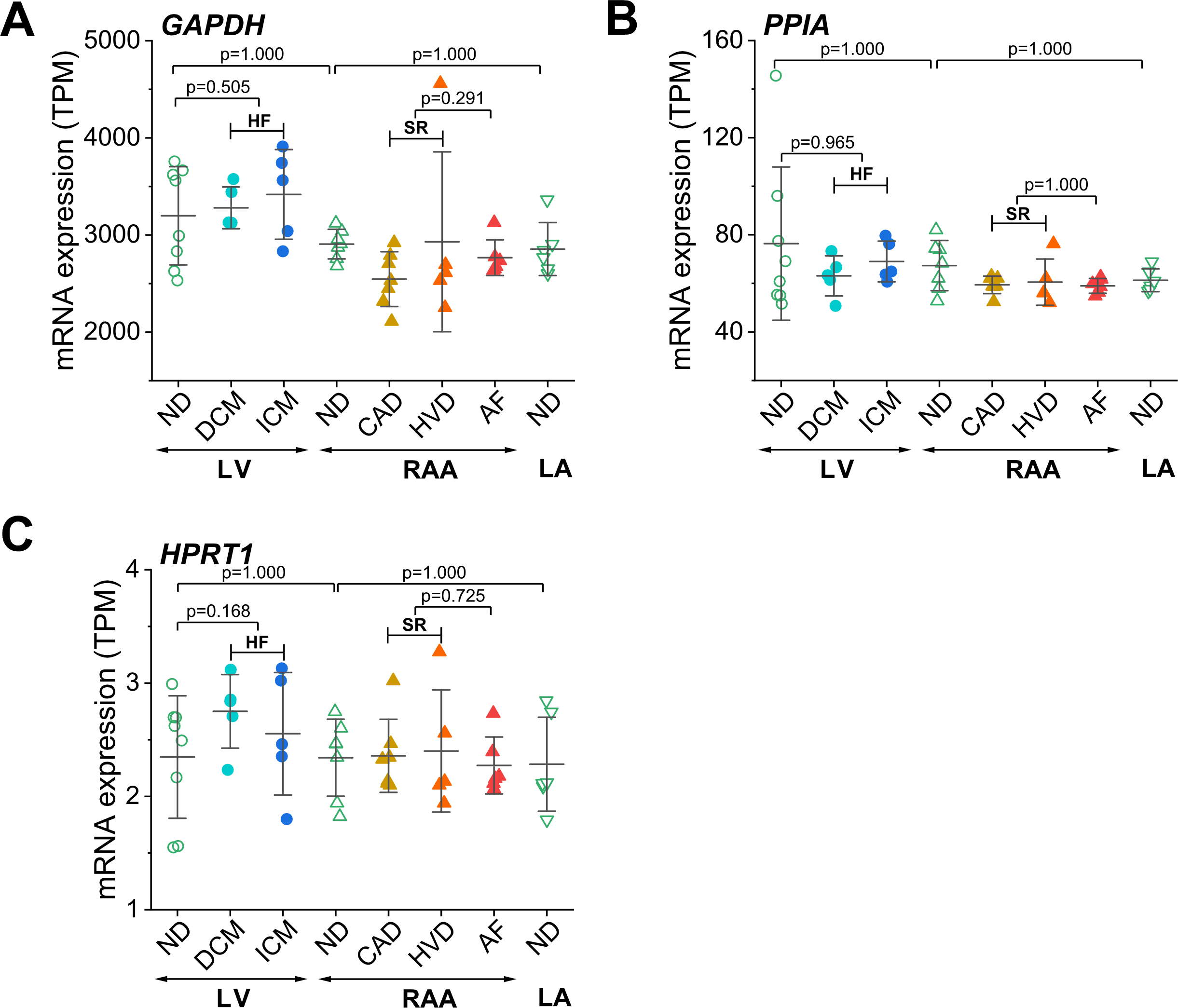

Supplement: Supplementary Figure 2 — mRNA expression of housekeeping genes in transcripts per kilobase million (TPM) assessed by RNA-seq. (A) GAPDH (glyceraldehyde-3-phosphate dehydrogenase), (B) PPIA (peptidylprolyl-isomerase A), and (C) HPRT1 (hypoxanthine-phosphoribosyltransferase 1). Sample provenance: left ventricle (LV; •, right atrial appendage (RAA; ▲), left atrium (LA; ▼). Patient’s health status: non-diseased (ND; green, open symbols); heart failure (HF): pooled data from dilated cardiomyopathy (DCM; light blue) and ischemic cardiomyopathy (ICM; blue); sinus rhythm (SR): pooled data from coronary artery disease (CAD; ocher) and heart valve disease (HVD; orange); atrial fibrillation (AF; red). The mean ± SD data are represented. Statistical significance was assessed by Dunn’s test. For HF and SR, statistical significance was assessed by the Mann–Whitney test. [file Image_2.tif]
